# Crisscross Moss Growth Optimization: An Enhanced Bio-Inspired Algorithm for Global Production and Optimization

**DOI:** 10.3390/biomimetics10010032

**Published:** 2025-01-07

**Authors:** Tong Yue, Tao Li

**Affiliations:** School of Geosciences, Yangtze University, Wuhan 430100, China; yuetong_ytu@163.com

**Keywords:** moss growth optimization, global optimization, reservoir production optimization, crisscross, metaheuristic, bionic algorithm

## Abstract

Global optimization problems, prevalent across scientific and engineering disciplines, necessitate efficient algorithms for navigating complex, high-dimensional search spaces. Drawing inspiration from the resilient and adaptive growth strategies of moss colonies, the moss growth optimization (MGO) algorithm presents a promising biomimetic approach to these challenges. However, the original MGO can experience premature convergence and limited exploration capabilities. This paper introduces an enhanced bio-inspired algorithm, termed crisscross moss growth optimization (CCMGO), which incorporates a crisscross (CC) strategy and a dynamic grouping parameter, further emulating the biological mechanisms of spore dispersal and resource allocation in moss. By mimicking the interwoven growth patterns of moss, the CC strategy facilitates improved information exchange among population members, thereby enhancing offspring diversity and accelerating convergence. The dynamic grouping parameter, analogous to the adaptive resource allocation strategies of moss in response to environmental changes, balances exploration and exploitation for a more efficient search. Key findings from rigorous experimental evaluations using the CEC2017 benchmark suite demonstrate that CCMGO consistently outperforms nine established metaheuristic algorithms across diverse benchmark functions. Furthermore, in a real-world application to a three-channel reservoir production optimization problem, CCMGO achieves a significantly higher net present value (NPV) compared to benchmark algorithms. This successful application highlights CCMGO’s potential as a robust and adaptable tool for addressing complex, real-world optimization challenges, particularly those found in resource management and other nature-inspired domains.

## 1. Introduction

Optimization is a crucial step in diverse fields such as engineering and economics, aiming to identify the optimal solution within a vast decision space [1]. This process is essential in practical applications to enhance efficiency and minimize costs. Many real-world tasks, including structural optimization in engineering, experimental parameter tuning in scientific research, and resource scheduling in industrial operations, often translate into complex optimization challenges [2]. These problems are frequently characterized by high-dimensional, nonlinear, and multimodal landscapes, often compounded by intricate constraints and dynamic environments [3].

Traditional optimization methods, such as gradient-based algorithms, simplex methods [4], and dynamic programming [5], have been extensively utilized. Gradient-based algorithms, including steepest descent [6] and conjugate gradient methods [7], are effective for convex and differentiable problems but struggle with nonconvexity, discontinuity, or the presence of multiple local optima. Simplex methods and dynamic programming perform well for specific problem types but typically suffer from the curse of dimensionality when addressing large-scale or high-dimensional problems [8]. Consequently, traditional optimization methods often fall short in addressing the complexities of many practical scenarios [9].

Metaheuristic algorithms have emerged as powerful alternatives, gaining significant attention for their adaptability and global search capabilities [10]. Unlike traditional methods, metaheuristic algorithms are not constrained by problem-specific properties such as convexity or differentiability. They employ stochastic mechanisms and principles inspired by natural phenomena to explore solution spaces effectively, making them particularly well suited for tackling complex, multimodal, and high-dimensional optimization problems [11].

In the literature, metaheuristic algorithms are generally classified into two main categories: evolutionary algorithms (EAs) and swarm intelligence (SI) algorithms [12]. Both EAs and SI share a similar framework structure: they initialize a set of solutions, iteratively update this population through specific operators, and return the current optimal solution when a termination condition is met. EAs are inspired by the principles of natural selection and biological evolution [13]. These algorithms iteratively refine a population of candidate solutions through selection, crossover, and mutation operators. Representative EAs include genetic algorithms (GAs) [14], differential evolution algorithms (DEs) [15], and spherical evolutionary algorithms (SEs) [16]. Swarm intelligence (SI) algorithms, conversely, draw inspiration from the collective behavior of social organisms such as birds, ants, and bees. These algorithms emphasize cooperation and information sharing among individuals in the population, leading to an efficient exploration of the search space and rapid convergence. Prominent SI algorithms include particle swarm optimization (PSO) [17], which models the social foraging behavior of bird flocks; ant colony optimization (ACO) [18], a method widely used for combinatorial problems such as routing and scheduling; and artificial bee colony (ABC) [19], which mimics the foraging behavior of honeybees.

Despite the success of metaheuristics in addressing complex optimization problems, these algorithms face fundamental limitations, as highlighted by the no free lunch (NFL) theorem [20]. This theorem fundamentally states that no single optimization algorithm is universally superior across all possible optimization problems. Instead, it reveals that algorithm performance is inherently problem-dependent. A strategy that excels at solving one type of problem may perform poorly when applied to a different type. This means there are always trade-offs in algorithm design. Consequently, understanding specific problem characteristics and tailoring algorithm design or improvements to those characteristics becomes crucial for achieving superior practical performance.

One prominent application domain where metaheuristic algorithms have demonstrated significant potential is reservoir production optimization [21]. In oil reservoir development, establishing an effective production scheme is essential for efficient hydrocarbon recovery and sustained production [22]. Optimizing injection and production processes in oil reservoirs involves considering numerous dynamic factors, such as reservoir heterogeneity, fluid properties, and operational constraints [23]. Achieving an optimal balance in injection rates and production strategies is vital for maximizing hydrocarbon recovery and minimizing operational costs. The inherent complexities of this domain, characterized by nonlinearity, uncertainty, and dynamic behavior, present significant challenges for traditional optimization methods. These methods often struggle with the high dimensionality and multifaceted nature of reservoir management problems. Consequently, there is a growing interest in applying metaheuristic algorithms, which offer adaptability and global search capabilities, to address these challenges and provide robust solutions for reservoir production optimization.

In the academic pursuit of optimizing petroleum injection and production, a variety of methodologies have been employed. Table 1 offers a review of some pertinent research in recent times. Although a substantial amount of research has concentrated on the development of surrogate models, frequently resorting to differential evolution (DE) or particle swarm optimization (PSO) as the optimization frameworks, it is of utmost significance that the optimizer chosen is strategically selected to match the particular traits of the problem. Such a tailored approach is vital for attaining superior optimization outcomes, as it allows for a more precise alignment with the unique demands and characteristics inherent in petroleum injection and production optimization, thus enhancing the potential for achieving enhanced efficiency and performance in this domain.

The moss growth optimizer (MGO), a swarm intelligence optimization algorithm introduced by Zheng et al. [30], draws inspiration from the growth patterns of moss in natural environments. MGO determines the evolutionary direction of the population through a “definite wind direction” mechanism. It then employs spore dispersal search and dual reproduction search for exploration and exploitation of the search space, respectively. Finally, a cryptobiosis mechanism modifies the conventional approach of directly altering individual solutions commonly used in metaheuristic algorithms. While experimental results have demonstrated the effectiveness of MGO, several limitations remain. Firstly, the algorithm lacks inter-population information exchange, leading to a suboptimal utilization of population knowledge. Secondly, the absence of dynamic adaptive strategies hinders the algorithm’s ability to optimize effectively across the different stages of the search process.

In this paper, we propose an improved MGO method, named CCMGO. By incorporating a crisscross strategy, CCMGO enhances inter-population information exchange, promotes offspring diversity, and accelerates convergence towards the optimal solution. Furthermore, the proposed dynamic grouping parameters balance the algorithm’s exploration and exploitation capabilities, enabling its effective adaptation across different optimization stages. The key contributions of this paper are threefold as follows:

- An improved MGO algorithm is proposed, which integrates a (CC) strategy and dynamic grouping parameters to enhance search efficiency and solution quality.
- CCMGO’s performance is rigorously evaluated against nine classic metaheuristic algorithms using the CEC2017 benchmark suite [31]. Statistical validation of the experimental results is conducted using the Wilcoxon and Friedman tests;
- CCMGO is applied to the practical problem of reservoir production optimization, utilizing a three-phase numerical simulation model. The net present value (NPV) achieved by CCMGO is compared to that of other optimization algorithms to demonstrate its efficacy in a real-world scenario.

This paper is structured into six sections. Section 1 provides an introduction, outlining the research background, motivation, and rationale for improving the MGO algorithm. Section 2 presents a detailed review of the original MGO algorithm, discussing its core principles and identifying areas for improvement. Section 3 introduces the proposed CCMGO algorithm, elaborating on the crisscross strategy and dynamic grouping parameters designed to enhance its performance. Section 4 details the experimental setup, presents the results of the comparative analysis with nine classic metaheuristic algorithms, and discusses the statistical validation of these results. Section 5 demonstrates the application of CCMGO to oil reservoir production optimization, showcasing its performance in comparison to other algorithms. Finally, Section 6 summarizes the key findings, discusses the advantages and limitations of CCMGO, and suggests potential avenues for future research.

## 2. The Original MGO

MGO is a metaheuristic algorithm proposed in 2024, which was inspired by the process of moss growth in natural environments, by Zheng et al. [30]. The MGO algorithm begins by establishing the evolutionary trajectory of the population through a mechanism known as the determination of wind direction, which utilizes a method of partitioning the population. Inspired by the processes of asexual, sexual, and vegetative reproduction in moss, the algorithm introduces two innovative search strategies: spore dispersal search for exploration and dual propagation search for exploitation. Lastly, the cryptobiosis mechanism is employed to modify the traditional metaheuristic approach of directly altering individuals’ solutions, thereby preventing the algorithm from becoming ensnared in local optima. By emulating the biological behavior of moss, the primary mathematical model of the MGO algorithm is structured as follows:

**1. Determination of wind direction:** MGO has introduced an innovative mechanism known as “Determining Wind Direction”, which establishes the evolutionary trajectory for all members of a population based on the spatial relationship between the majority of individuals and the optimal individual.

Firstly, the grouping operation is performed for each dimension of the population X, and the optimal individual in the current population is $M_{best}$, and the jth dimension of $M_{best}$ is used as the threshold; $X_{i,j}$ which is greater than $M_{best,j}$ belongs to the $DX_{j1}$ set, and $X_{i,j}$ which is less than $M_{best,j}$ belongs to the $DX_{j2}$ set, and after that, the determined set is selected as ${divX}_{j}$ according to Equation (1).

(1) $${divX}_{j}=\left\{\begin{array}{cc} {DX}_{j1}, & count({DX}_{j1})\geq count({DX}_{j2})\\ {DX}_{j2}, & count({DX}_{j1})<count({DX}_{j2}) \end{array}\right.$$

where count(·) means counting the number of elements in a given set. This dividing process will be performed several times and the set after dividing is as follows:

(2) $$di\nu X=\{M_{i}=(M_{i,1},M_{i,2},\ldots ,M_{i,dim})|M_{i}\in \overset{dn}{\underset{j=1}{⋂}}\;di\nu X_{p_{j}},M_{i}\in X\}$$

where dn denotes the number of times it has been dividing, and $p_{j}$ denotes a randomly selected dimension, which simply means that the set is first determined to be divided dn times, followed by randomly selecting dn dimensions, after which the first dimension is used for the dividing the set, and after that the divided set is used for the second divided using the second dimension selected, and so on until the number of times the dividing is exhausted, to obtain the diνX. Based on the above information, the wind direction vectors are as follows:

(3) $$D_{-}wind=M_{best}-mean(divX)$$

where $D_{-}wind$ denotes $M_{best}$ minus a vector that averages each dimension of divX;

**2. Spore dispersal search:** The exploration stage of MGO simulates the dissemination process of spores. In the presence of strong winds, the spores can travel longer distances, whereas in turbulent conditions, the dispersion distance of the spores is shorter. The modeling of this process is shown in Equation (4).

(4) $$M_{i}^{new}=\{\begin{array}{cc} M_{i}+step1\cdot D_{-}wind & r_{1}>0.2\\ M_{i}+step2\cdot D_{-}wind & r_{1}\leq 0.2 \end{array}$$

where $M_{i}^{new}$ denotes the new individual generated by the i^th^ individual, and $r_{1}$ is a random number between 0 and 1. step1 and step2 represent the update steps for the two modes, respectively, as shown in Equations (5) and (6).

(5) $$step1=w\cdot \left(r_{2}-0.5\right)\cdot E$$

where w is a constant value of two; $r_{2}$ is a random vector between zero and one, and E denotes the strength of the wind as shown in Equation (7).

(6) $$step2=0.1\cdot w\cdot (r_{3}-0.5)\cdot E\cdot [1+\frac{E}{2}\cdot (1+tanh\beta \sqrt{1-\beta^{2}})]$$

where $r_{3}$ is a random vector between 0 and 1, and β is shown in Equation (8).

(7) $$E=1-\frac{FEs}{MaxFEs}$$

where FEs denotes the current number of evaluations, and MaxFEs denotes the maximum number of evaluations.

(8) $$\beta =\frac{count\left(divX\right)}{count\left(X\right)}$$

where β denotes the proportion of the size of divX and X;

**3. Dual propagation search:** The dual propagation search strategy simulates the phenomenon of sexual and asexual reproduction of spores, and it is important to note that the MGO algorithm uses dual propagation search with 80% probability, which is mathematically modeled as shown below:

(9) $$\left.\left\{\begin{array}{l} M_{i}^{new}=(1-act)\cdot M_{i}+act\cdot M_{best}\;r_{4}>0.5\\ M_{i,j}^{new}=M_{best,j}+step3\cdot D\_wind_{j}\;\;r_{4}\leq 0.5 \end{array}\right.\right.$$

In Equation (9), the part where $r_{4}>0.5$ means it is updating the ith individual, while the part where $r_{4}\leq 0.5$ means it is updating the jth dimension of the ith individual, where act denotes the parameter controlling whether $M_{best}$ is utilized or not as shown in Equation (10), and step3 denotes the update step as shown in Equation (11).

(10) $$act=act=\left\{\begin{array}{c} 1,\;\;\frac{1}{1.5-10\cdot r_{5}}\geq 0.5\\ 0,\;\;\frac{1}{1.5-10\cdot r_{5}}<0.5 \end{array}\right.$$

where $r_{5}$ is a random vector between zero and one.

(11) $$step3=0.1\cdot \left(r_{6}-0.5\right)\cdot E$$

where $r_{6}$ is a random vector between zero and one, and E denotes the strength of the wind as in Equation (7);

**4. Cryptobiosis mechanism:** The cryptobiosis mechanism, employed to replace the greedy selection component of traditional metaheuristic algorithms, draws inspiration from the cryptobiosis observed in mosses—an extraordinary ability enabling these organisms to recover and thrive following periods of dormancy or desiccation. Unlike the conventional greedy selection, this mechanism uses an archive to systematically store updated individuals across generations. When specific conditions, such as reaching a maximum record count, are satisfied, the selection process is triggered to replace the current individual with the best performer from the archive. This strategy allows each individual to initiate their search from the same location multiple times, thereby significantly enhancing the optimization performance of the MGO. The interested readers are referred to its detailed exposition in the study by [30].

**Figure 1:**
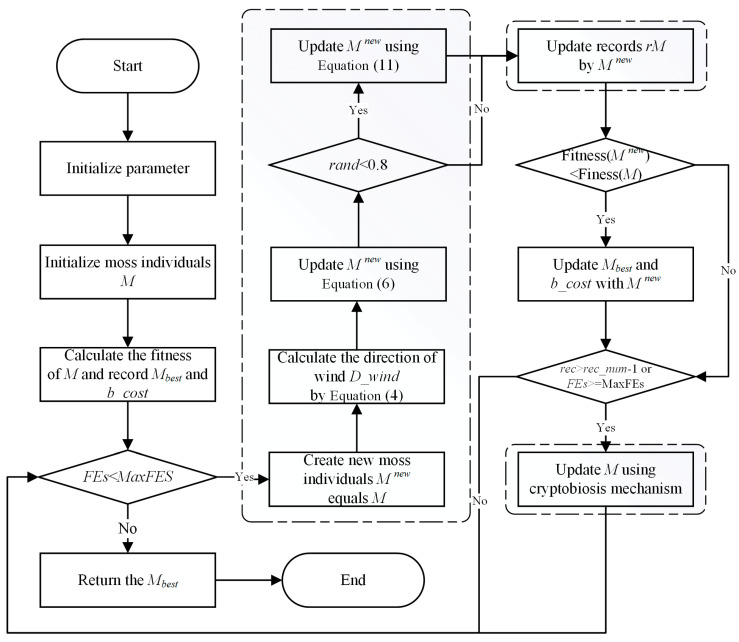
Flowchart of the MGO.

In summary, MGO starts by randomly generating a set of initial individuals, and during each iteration, it first determines the evolutionary direction of the population based on the wind direction, after which it performs spore dispersal search and dual propagation search sequentially based on the given probability and updates each individual through the cryptobiosis mechanism. This process is repeated until the maximum number of evaluations is reached and the optimal individual of the current population is returned. The flowchart of MGO is shown in Figure 1.

## 3. Proposed CCMGO

### 3.1. Crisscross Strategy

The CC concept, originating from the crisscross optimization (CSO) algorithm introduced in 2014 [32], comprises two key components: horizontal crossover search (HCS) and vertical crossover search (VCS). CC embodies the principle of “The Doctrine of the Mean”, dynamically adjusting the search trajectory within the solution space. HCS facilitates information exchange among individuals, while VCS enables intersection across different dimensions of the same individual. This unique intersection strategy, coupled with a competitive selection mechanism, enhances the algorithm’s global search capabilities and accelerates convergence.

In this study, we integrate the CC strategy into the original MGO algorithm to enhance inter-population information dissemination, thereby facilitating a more effective evasion of local optima. The HCS and VCS strategies, as incorporated within CC, are detailed below.

#### 3.1.1. Horizontal Crossover Search

The HCS operation involves randomly selecting particles from the population and pairing them to perform a crossover operation. This process leverages population information to enhance the algorithm’s exploratory capabilities. The mathematical formulation of HCS is defined in Equations (12) and (13).

(12) $$H_{i}^{j}=r_{1}\times x_{i}^{j}+\left(1-r_{1}\right)\times x_{k}^{j}+c_{1}\times \left(x_{i}^{j}-x_{k}^{j}\right)$$

(13) $$H_{k}^{j}=r_{2}\times x_{k}^{j}+\left(1-r_{2}\right)\times x_{i}^{j}+c_{2}\times \left(x_{k}^{j}-x_{i}^{j}\right)$$

where $r_{1}$ and $r_{2}$ denote random numbers within the interval [0, 1]; $c_{1}$ and $c_{2}$ denote random numbers within the interval [−1, 1]; $x_{i}^{j}$ denotes the value of the jth dimension of the ith particle, and $x_{k}^{j}$ is the value of the jth dimension of the kth particle. $H_{i}^{j}$ and $H_{k}^{j}$ are the new offspring generated by the HCS from the two particles. HCS then proceeds with a greedy selection to preserve the fittest individuals between the offspring and their parents.

#### 3.1.2. Vertical Crossover Search

The VCS operation entails randomly selecting dimensions within each particle and pairing them for a crossover operation. This mechanism utilizes individual information to enhance the algorithm’s exploitative capabilities. The specific formulation of the VCS operation is detailed in Equation (14)

(14) $$V_{i}^{j}=r_{3}\times x_{i}^{j1}+\left(1-r_{3}\right)\times x_{i}^{j2}$$

where $r_{3}$ denotes a random number within the interval [0, 1]; $x_{i}^{j1}$ and $x_{i}^{j2}$ denote the values of the two dimensions randomly picked by the ith individual; $V_{i}^{j}$ denotes the offspring generated by the VCS operation. Analogous to HCS, VCS employs a greedy selection strategy, retaining individuals with superior fitness values from the offspring and their progenitors.

### 3.2. Dynamic Population Divisions Parameter

The direction determination phase in the original MGO algorithm necessitates specifying the number of population divisions, which subsequently determines the final direction vectors as defined in Equations (2) and (3). In the original MGO, this number is fixed at $\frac{dim}{4}$, a setting that may not adequately address the algorithm’s requirements across various stages of the optimization process.

Intuitively, during the initial exploration phase, a larger proportion of the population should contribute to determining the update direction, implying fewer population divisions. Conversely, in the later exploitation phase, it is preferable for a smaller subset of individuals to influence the update direction, necessitating a larger number of population divisions. To address this, we propose a dynamic population division parameter that adjusts the number of divisions in each iteration. This ensures that the algorithm’s update direction is influenced by an appropriate number of individuals at each stage, thereby enhancing optimization efficiency. The formulation of this parameter is as follows:

(15) $$divide\_num=(\frac{FEs}{MaxFEs}+1)\cdot \frac{dim}{4}$$

where FEs is the current number of evaluations; MaxFEs is the maximum number of evaluations, and dim is the dimension of the problem.

### 3.3. The Proposed CCMGO

**Figure 2:**
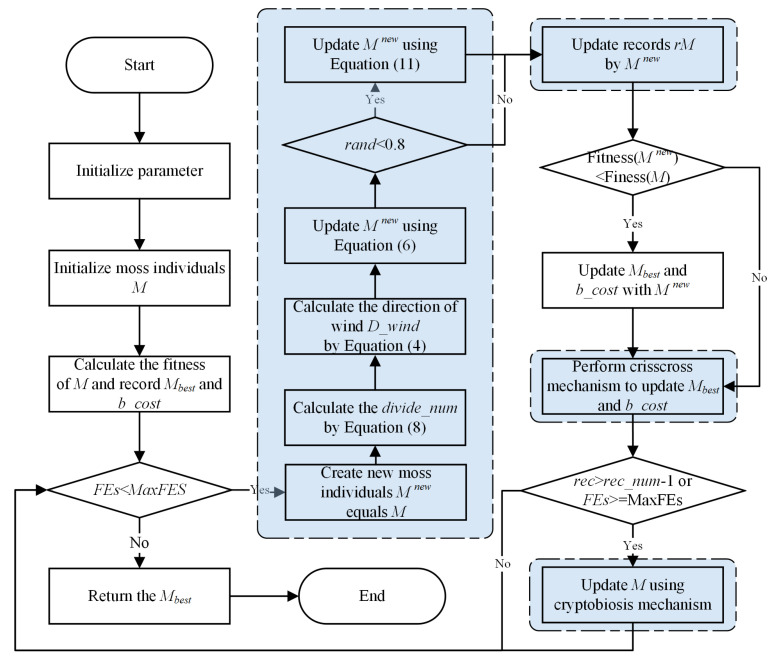
Flowchart of the CCMGO.

This section introduces the proposed CCMGO algorithm. First, CCMGO initializes the MGO parameters and generates the initial population. Then, the algorithm proceeds with population updating using the original MGO framework. In each iteration, the number of divisions for determining the wind direction vector is dynamically adjusted. Before concluding each iteration, the CC strategy is applied to generate a new population. This iterative process continues until the predefined maximum number of iterations is reached. The algorithm’s workflow is illustrated in Figure 2.

Algorithm 1 provides the pseudo-code for the CCMGO.
**Algorithm 1** Pseudo-code of the CCMGOSet parameters: the maximum evaluation number MaxFEs, the problem dimension dim, and the population size N.
Initialize population M
FEs = 0 **For** i=1:N
   **Evaluate** the fitness value of $M_{i}$
  **Find** the global min $M_{best}$ and fitness b_cost **End** For **While (**FEs<MaxFEs)
   **Calculate** the divide_num by Equation (15)   /* Dynamic Divisions Parameter */
   **Calculate** the wind direction D_wind by Equation (3)   **For** i=1:N
    **Create** the new search agent $M_{i}^{best}$ equals $M_{i}$
    **Update** the $M_{i}^{best}$ by Equation (4)    **If** rand<0.8
      **Update** $M_{i}^{new}$ by Equation (9)    **End if**    **If** $Fitness\left(M_{i}^{new}\right)<Fitness(M_{best})$
      $M_{best}=M_{i}^{new}$
      $b\_cost=Fitness(M_{i}^{new})$
    **End if**   **End for**   **For** i=1:N
    **Update** $M_{i}$ using the cryptobiosis mechanism   **End for**   FEs=FEs+N
   **For** i=1:N                                      /*CC*/    **Perform** Horizontal crossover search to update $M_{i}$
    **Perform** Vertical crossover search to update $M_{i}$
    **Update** $M_{best}$
   **End**
For
   FEs=FEs+N
**End While**
**Return** $M_{best}$
**End**


Four primary factors contribute to the computational complexity of CCMGO: population initialization, fitness function evaluation, particle position updates, and the crisscross (CC) strategy. Therefore, the overall complexity can be expressed as O(CCMGO) ≈ O(T × N) + O(T × N) + O(T × N × D) + O(T × N × D), which simplifies to O(T × N × D).

## 4. Experimental Results and Analysis

This section evaluates the performance of the proposed CCMGO algorithm using the 29 benchmark functions from the CEC2017 test suite. Experiments are conducted under fair conditions using these industry-standard benchmarks. The experimental setup consists of an Intel i5-13600KF (Intel Corporation, Santa Clara, CA, USA) processor with 32 GB of RAM, running the Windows 11 operating system. The algorithms are implemented in MATLAB 2024a. For the comparative analysis, all algorithms utilize a population size of 30, a problem dimension of 30, and a maximum of 300,000 function evaluations. Each function is executed 30 times independently, and the average and standard deviation of the resulting objective function values are reported.

### 4.1. Benchmark Functions Overview

This subsection describes the 29 benchmark functions from the 2017 IEEE Congress on Evolutionary Computation (CEC2017) test suite [31]. These functions are categorized into four groups: unimodal, multimodal, hybrid, and composition functions. This diverse set of functions allows for a comprehensive evaluation of the algorithm’s performance across various function landscapes. Table 2 provides a summary of the CEC2017 benchmark functions.

### 4.2. Performance Comparison with Other Algorithms

This section presents a comparative performance evaluation of the proposed CCMGO algorithm against nine widely used metaheuristic optimization algorithms using the CEC2017 benchmark functions. The comparative algorithms include MGO [30], WOA [33], GWO [34], MFO [35], SCA [36], PSO [37], SMA [38], BA [39], and FA [40]. All algorithms were implemented following the descriptions provided in their respective original papers to maintain methodological consistency. To ensure a fair comparison, the hyperparameter settings for each comparative algorithm were configured according to the recommendations in their original studies (as summarized in Table 3) and were not adjusted further. This means that we used the most commonly reported parameter values from their original papers and other relevant literature, providing an impartial setting for each method.

Table 4 provides a comprehensive performance comparison of CCMGO and the other nine algorithms on the CEC2017 benchmark functions. The table reports the average (Avg) and standard deviation (Std) of the fitness values for each algorithm on each benchmark function. The average value provides a measure of the overall performance of each method on each problem, and the standard deviation shows the stability of the approach when repeated. Using these two metrics provides a robust method for measuring performance. Furthermore, the Friedman test ranks (Rank) of each algorithm are presented, along with the average rank (Avg) across all functions. The Friedman test was chosen as it is a non-parametric statistical test suitable for comparing the performance of multiple algorithms across a number of problems. The “+/−/=” column denotes instances where CCMGO outperformed (+), matched (=), or underperformed (−) compared to the other algorithms. This pairwise comparison allows us to specifically see where our proposed approach shows an improvement over other standard benchmarks. By including all of these different metrics, we are able to make a rigorous and detailed assessment of the performance of the proposed method.

The results in Table 4 demonstrate that CCMGO achieves the best overall performance, attaining the lowest average rank of 1.6207 among all algorithms on the CEC2017 benchmark suite. Specifically, CCMGO outperforms MGO, the second-best performing algorithm with an average rank of 2.6552, on 18 out of 29 functions. This superior performance and lower average rank highlight CCMGO’s enhanced optimization reliability and effectiveness in tackling complex optimization problems. The specific performance of CCMGO in comparison to the other benchmark algorithms, combined with the lower average rank of our approach, demonstrates the validity and improved performance of CCMGO compared to other similar methods. This suggests that our approach can be generalized to other problem domains and may provide improvements over other methods.

Table 5 further substantiates the superior performance of CCMGO on the CEC2017 benchmark suite. The Wilcoxon signed-rank test results, presented in Table 5, reveal statistically significant differences (*p* < 0.05) between CCMGO and the other algorithms for most of the benchmark functions. These findings strongly support the conclusion that CCMGO offers significantly better optimization performance compared to the other algorithms, reinforcing its reliability and effectiveness for challenging optimization tasks.

**Figure 3:**
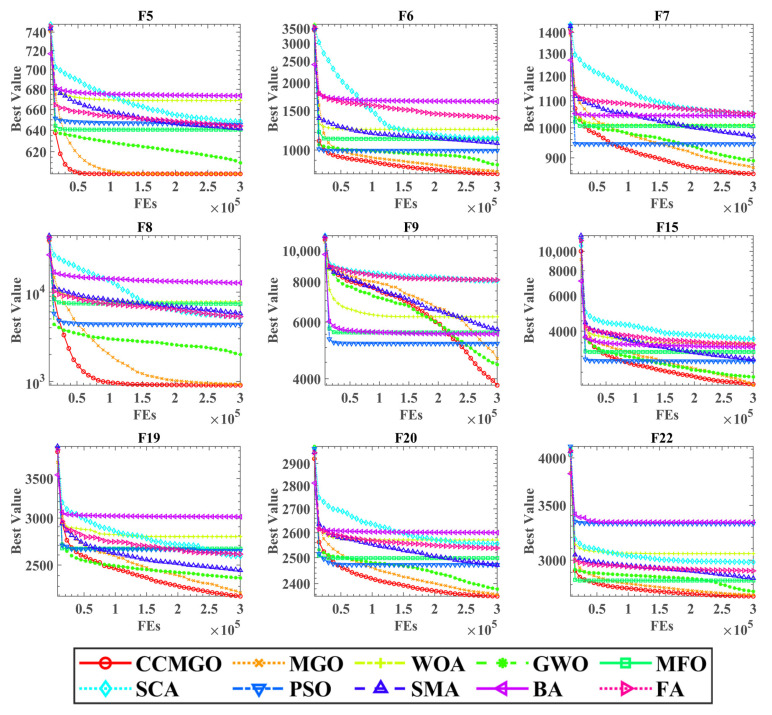
Convergence curves of the CCMGO on benchmarks with other algorithms.

Figure 3 illustrates the convergence curves of CCMGO compared to the other nine algorithms on the benchmark functions. The horizontal axis represents the number of function evaluations, while the vertical axis represents the best fitness value achieved.

The convergence curves clearly demonstrate that CCMGO consistently achieves lower fitness values compared to the other algorithms across the benchmark functions. This indicates that CCMGO converges to better solutions more efficiently, requiring fewer function evaluations to reach comparable or superior fitness levels. This observation highlights CCMGO’s effective exploration and exploitation of the search space and its ability to avoid premature convergence to local optima. In summary, the incorporation of the crisscross strategy significantly enhances the search performance of MGO, enabling it to outperform competing algorithms on the benchmark functions.

## 5. Application to Production Optimization

The objective of reservoir production optimization is to determine the optimal control settings for each well to maximize NPV. However, the large number of wells and production cycles leads to a combinatorial explosion of possible solutions, resulting in a high-dimensional, NP-hard optimization problem. This complexity makes evolutionary algorithms well suited for tackling such challenges. In this study, we use the Eclipse reservoir simulator to apply CCMGO to a three-channel reservoir model and compare its performance against several widely used metaheuristic algorithms. For this study, nonlinear constraints associated with oilfield production are disregarded, and the NPV, as defined in Equation (16), serves as the objective function.

(16) $$NPV\left(x,z\right)=\sum_{t=1}^{n}\;\Delta t\frac{Q_{o,t}\cdot r_{o}-Q_{w,t}\cdot r_{w}-Q_{i,t}\cdot r_{i}}{{\left(1+b\right)}^{p_{t}}}$$

where x denotes the set of variables to be optimized; z denotes the state parameter of the model; n denotes the total simulation time; $Q_{o,t}$, $Q_{w,t}$, and $Q_{i,t}$ are the rates of oil production, water production, and water injection, respectively. $r_{o}$ denotes the oil revenue; $r_{w}$ and $r_{i}$ denote the cost of treating and injecting the water, respectively; b is the annual interest rate, and $p_{t}$ is the number of years that have passed.

### 5.1. Three-Channel Model

The three-channel reservoir model is a typical non-homogeneous two-dimensional reservoir, which has four injection wells as well as nine production wells arranged in a five-point pattern. This model is shaped with a grid consisting of 625 grid blocks, where each grid side is 100 ft; each grid block is 20 ft thick, and all grid blocks have a porosity of 0.2, as shown in Table 6. These are all standard values that have been used in other studies and can be considered typical values for this kind of problem. To ensure our results are comparable with other studies in the literature, we used the same values as in reference [24].

The optimization variables in this production optimization problem are the injection rate for each injection well and the fluid recovery rate for the production wells. The water injection rate ranges from 0 to 500 STB/DAY, and the water extraction rate for the production wells ranges from 0 to 200 STB/DAY. Thermal storage is utilized for 1800 days, with a decision time step of 360 days. As a result, the decision variable has a dimensionality of 65.

NPV is the fitness function for this optimization problem and is determined by various parameters, including the oil price (80.0 USD/STB), the water injection cost (5.0 USD/STB), and the water treatment cost (5.0 USD/STB). To simplify the model, it is assumed that the interest rate per annum is 0%.

### 5.2. Analysis and Discussion of the Experimental Results

This subsection provides a detailed analysis of the experimental results for optimizing the three-channel oil reservoir production model. The performance of CCMGO is compared with six other optimization algorithms: MGO, MFO, GWO, PSO, SMA, and WOA. All algorithms were repeated five times, and the metrics such as the mean, standard deviation (Std), the best and worst values of the objective function, and the experimental results of CCMGO are presented and discussed.

Table 7 summarizes the experimental results of each algorithm, including metrics such as the mean, standard deviation, and the best and worst values. CCMGO achieves the highest mean value (9.4969 × 10^7^) among all algorithms, highlighting its superior ability to consistently deliver high-quality solutions. Moreover, its standard deviation (1.7636 × 10^6^) is relatively low compared to MFO (3.2705 × 10^6^) and GWO (3.8653 × 10^6^), indicating greater stability and reliability. These results demonstrate CCMGO’s effectiveness in exploring and exploiting the search space and its robustness in maintaining consistent performance under various conditions.

**Figure 4:**
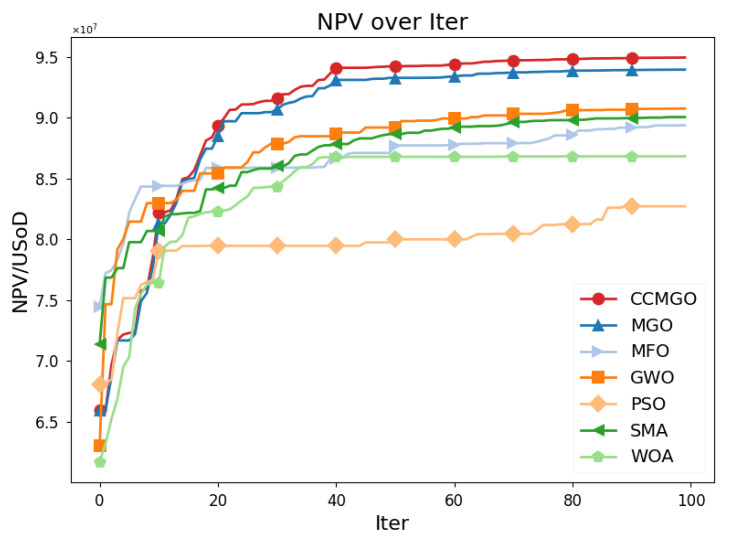
Convergence of NPV values for the different algorithms over iterations.

Figure 4 shows the NPV progression of CCMGO and six other algorithms over 100 iterations, with the horizontal axis representing the iteration count and the vertical axis showing the corresponding NPV values.

CCMGO exhibits the fastest convergence and consistently achieves the highest NPV among all algorithms. It rapidly approaches its peak performance within the first 30 iterations and maintains a clear advantage throughout the optimization process. In contrast, MGO converges more slowly and achieves slightly lower NPV values than CCMGO by the end of the iterations. The other algorithms, including MFO, GWO, SMA, PSO, and WOA, show varying degrees of suboptimal performance. MFO, GWO, and SMA achieve moderate results, while PSO and WOA converge significantly slower and yield lower final NPV values.

In summary, the results demonstrate the effectiveness of CCMGO in optimizing the oil reservoir production model. CCMGO achieves the highest NPV and converges faster than the other algorithms, confirming its superior performance.

## 6. Conclusions

This study introduced CCMGO, an enhanced MGO algorithm incorporating a CC strategy and a dynamic population divisions parameter. The CC strategy promotes information exchange among individuals within the population, thereby increasing offspring diversity and accelerating convergence towards optimal solutions. The dynamic population divisions parameter balances exploration and exploitation, leading to a more efficient search process.

The performance of CCMGO was rigorously evaluated using the CEC2017 benchmark suite and compared against nine prominent metaheuristic optimization algorithms. Experimental results demonstrated that CCMGO consistently outperformed the other algorithms across a variety of benchmark functions, achieving a significantly lower average rank of 1.6207 compared to MGO’s 2.6552 on the CEC2017 suite. Furthermore, CCMGO outperformed MGO on 18 out of 29 functions in these tests. Furthermore, CCMGO was applied to a real-world reservoir production optimization problem, where it achieved a higher NPV and the highest mean value of 9.4969 × 10^7^ compared to all other algorithms, while also maintaining a relatively low standard deviation of 1.7636 × 10^6^, demonstrating superior performance and stability. This practical application illustrates CCMGO’s potential for solving complex, real-world optimization challenges.

Future research will focus on enhancing the scalability of CCMGO for high-dimensional problems and extending its capabilities to address multi-objective optimization tasks. We also plan to explore the application of CCMGO in a wider range of real-world optimization problems to further demonstrate its adaptability and effectiveness.

## Figures and Tables

**Table 1:** Recent years’ advances in optimization methodologies for petroleum production.

Name	Summary
SADE-Sammon [24]	Chen et al. proposed a new framework for oil reservoir production optimization, combining surrogate-assisted evolutionary algorithms and Sammon mapping for dimensionality reduction to improve the efficiency of expected NPV maximization. It outperformed classical evolutionary algorithms and other dimensionality reduction methods.
GLSADE [25]	Chen et al. proposed the global and local surrogate-model-assisted differential volution (GLSADE) for waterflooding production optimization. It refines the surrogate model to focus on promising regions, showing better NPV and faster convergence than traditional methods on benchmarks and real-world applications.
PSO, GA [26]	Bohorquez et al. used stochastic methods (GA and PSO) with surrogate models for FCCU multi-objective optimization. PSO outperformed GA in naphtha yield (3% increase) with fewer evaluations. Stochastic optimization was better than deterministic optimization for FCCU design and planning, aiding refinery profit and compliance.
GA-SAO [27]	Oliveira et al. proposed a hybrid optimization strategy for dynamic waterflooding management, maximizing NPV by optimizing well allocation rates and switching times. This approach synergistically combined GA for global search with sequential approximation optimization (SAO) for local refinement, effectively identifying optimal well management strategies and demonstrating that increased operational flexibility enhances NPV. The incorporation of cycle duration variables reduced the dimensionality of the design space while maintaining recovery efficiency.
CSDE [28]	Zhang et al. proposed CSDE for waterflooding optimization, handling nonlinear inequality constraints. A two-stage method with SVM for feasible solution identification and RBF surrogate model for objective function approximation was used. CSDE improved efficiency and NPV, surpassing traditional and single-model evolutionary algorithms.
CCWFO [29]	Zhao et al. developed an enhanced water flow optimizer (CCWFO) by incorporating a cross-search strategy to accelerate convergence and improve the accuracy of the original water flow optimizer (WFO). Evaluated against CEC2017 benchmarks, CCWFO demonstrated superior global optimization capabilities compared to other metaheuristic algorithms. The application of CCWFO to a three-channel reservoir model yielded a higher NPV within equivalent evaluation limits, establishing it as a robust alternative to classical evolutionary algorithms for reservoir production optimization.

**Table 2:** CEC2017 benchmark functions.

Function	Function Name	Class	Optimum
F1	Shifted and Rotated Bent Cigar Function	Unimodal	100
F2	Shifted and Rotated Zakharov Function	Unimodal	300
F3	Shifted and Rotated Rosenbrock’s Function	Multimodal	400
F4	Shifted and Rotated Rastrigin’s Function	Multimodal	500
F5	Shifted and Rotated Expanded Schaffer’s F6 Function	Multimodal	600
F6	Shifted and Rotated Lunacek Bi-Rastrigin Function	Multimodal	700
F7	Shifted and Rotated Non-Continuous Rastrigin’s Function	Multimodal	800
F8	Shifted and Rotated Lévy Function	Multimodal	900
F9	Shifted and Rotated Schwefel’s Function	Multimodal	1000
F10	Hybrid Function 1 (N = 3)	Hybrid	1100
F11	Hybrid Function 2 (N = 3)	Hybrid	1200
F12	Hybrid Function 3 (N = 3)	Hybrid	1300
F13	Hybrid Function 4 (N = 4)	Hybrid	1400
F14	Hybrid Function 5 (N = 4)	Hybrid	1500
F15	Hybrid Function 6 (N = 4)	Hybrid	1600
F16	Hybrid Function 6 (N = 5)	Hybrid	1700
F17	Hybrid Function 6 (N = 5)	Hybrid	1800
F18	Hybrid Function 6 (N = 5)	Hybrid	1900
F19	Hybrid Function 6 (N = 6)	Hybrid	2000
F20	Composition Function 1 (N = 3)	Composition	2100
F21	Composition Function 2 (N = 3)	Composition	2200
F22	Composition Function 3 (N = 4)	Composition	2300
F23	Composition Function 4 (N = 4)	Composition	2400
F24	Composition Function 5 (N = 5)	Composition	2500
F25	Composition Function 6 (N = 5)	Composition	2600
F26	Composition Function 7 (N = 6)	Composition	2700
F27	Composition Function 8 (N = 6)	Composition	2800
F28	Composition Function 9 (N = 3)	Composition	2900
F29	Composition Function 10 (N = 3)	Composition	3000

**Table 3:** Hyperparameter settings of comparative algorithms.

Name	Parameters
CCMGO	w = 2; rec_num = 10; divide_num = [dim/4, dim]; d1 = 0.2
MGO	w = 2; rec_num = 10; divide_num = dim/4; d1 = 0.2;
WOA	$a_{1\;}$$=[2,\;0];\;a_{2}$ =[−1,−2]; b = 1
GWO	a = [2, 0]
MFO	b = 1; t = [−1, 1]; a = [−1, −2]
SCA	a = 2
PSO	$V_{max}$$\;=\;6;\;W_{max}$$\;=\;0.9,\;W_{min}$$\;=\;0.2;\;C_{1}$$\;=\;2;\;C_{2}$ = 2
SMA	a = [2, 0]; vb = [−2, 2]; E = [0, 2]
BA	Qmin = 0; Qmax = 2
FA	alpha = 0.5; betamin = 0.2; gamma = 1

**Table 4:** Results of the CCMGO and the other algorithms on CEC2017.

	F1		F2		F3	
	Avg	Std	Avg	Std	Avg	Std
CCMGO	9.3875 × 10^4^	1.2429 × 10^5^	6.3469 × 10^3^	1.8785 × 10^3^	4.9078 × 10^2^	1.4145 × 10^1^
MGO	1.6156 × 10^5^	2.8369 × 10^5^	4.7837 × 10^4^	9.1011 × 10^3^	4.9353 × 10^2^	1.1080 × 10^1^
WOA	3.1479 × 10^6^	2.1521 × 10^6^	1.5459 × 10^5^	6.5052 × 10^4^	5.5521 × 10^2^	4.3391 × 10^1^
GWO	2.0741 × 10^9^	1.1617 × 10^9^	3.1738 × 10^4^	1.1031 × 10^4^	5.9004 × 10^2^	1.0153 × 10^2^
MFO	9.3736 × 10^9^	7.0162 × 10^9^	8.0164 × 10^4^	5.6794 × 10^4^	1.3305 × 10^3^	8.6241 × 10^2^
SCA	1.2688 × 10^10^	2.4698 × 10^9^	3.6850 × 10^4^	5.6118 × 10^3^	1.4518 × 10^3^	2.4771 × 10^2^
PSO	3.1410 × 10^3^	4.1096 × 10^3^	3.0000 × 10^2^	2.1517 × 10^−3^	4.6319 × 10^2^	2.4383 × 10^1^
SMA	2.6843 × 10^9^	9.4623 × 10^8^	3.6783 × 10^4^	8.9412 × 10^3^	6.2258 × 10^2^	6.1424 × 10^1^
BA	5.4272 × 10^5^	2.8167 × 10^5^	3.0011 × 10^2^	1.1756 × 10^−1^	4.7692 × 10^2^	2.6001 × 10^1^
FA	1.4463 × 10^10^	1.7921 × 10^9^	5.9894 × 10^4^	6.6363 × 10^3^	1.3073 × 10^3^	1.4159 × 10^2^
	F4		F5		F6	
	Avg	Std	Avg	Std	Avg	Std
CCMGO	5.4730 × 10^2^	8.9643 × 10^0^	6.0000 × 10^2^	4.3867 × 10^−3^	7.8150 × 10^2^	9.7607 × 10^0^
MGO	5.6413 × 10^2^	8.5239 × 10^0^	6.0000 × 10^2^	1.2025 × 10^−4^	8.0114 × 10^2^	1.2546 × 10^1^
WOA	7.8507 × 10^2^	6.2600 × 10^1^	6.6882 × 10^2^	1.3300 × 10^1^	1.2357 × 10^3^	1.0049 × 10^2^
GWO	5.9923 × 10^2^	2.8902 × 10^1^	6.0988 × 10^2^	4.3030 × 10^0^	8.5780 × 10^2^	3.5598 × 10^1^
MFO	7.1971 × 10^2^	5.1124 × 10^1^	6.4053 × 10^2^	1.2079 × 10^1^	1.1219 × 10^3^	2.2063 × 10^2^
SCA	7.7487 × 10^2^	2.0651 × 10^1^	6.4852 × 10^2^	4.6381 × 10^0^	1.1262 × 10^3^	4.2682 × 10^1^
PSO	6.9375 × 10^2^	4.0546 × 10^1^	6.4544 × 10^2^	7.4126 × 10^0^	9.9383 × 10^2^	5.1109 × 10^1^
SMA	7.1472 × 10^2^	3.6475 × 10^1^	6.4161 × 10^2^	7.6309 × 10^0^	1.0714 × 10^3^	5.9583 × 10^1^
BA	8.4918 × 10^2^	5.4933 × 10^1^	6.7349 × 10^2^	9.6446 × 10^0^	1.6510 × 10^3^	1.8809 × 10^2^
FA	7.5784 × 10^2^	1.0742 × 10^1^	6.4407 × 10^2^	2.9626 × 10^0^	1.3864 × 10^3^	4.1624 × 10^1^
	F7		F8		F9	
	Avg	Std	Avg	Std	Avg	Std
CCMGO	8.5046 × 10^2^	7.1227 × 10^0^	9.0542 × 10^2^	5.6323 × 10^0^	3.8173 × 10^3^	3.8526 × 10^2^
MGO	8.6955 × 10^2^	1.3256 × 10^1^	9.3020 × 10^2^	2.2531 × 10^1^	4.6229 × 10^3^	3.8254 × 10^2^
WOA	1.0102 × 10^3^	5.4531 × 10^1^	7.7603 × 10^3^	2.2539 × 10^3^	6.2311 × 10^3^	6.9373 × 10^2^
GWO	8.9081 × 10^2^	2.3697 × 10^1^	2.0043 × 10^3^	6.3529 × 10^2^	4.4260 × 10^3^	1.3461 × 10^3^
MFO	1.0072 × 10^3^	4.3452 × 10^1^	7.4238 × 10^3^	2.0970 × 10^3^	5.5716 × 10^3^	8.0899 × 10^2^
SCA	1.0524 × 10^3^	1.6918 × 10^1^	5.3439 × 10^3^	7.4913 × 10^2^	8.0736 × 10^3^	3.8373 × 10^2^
PSO	9.4496 × 10^2^	2.5764 × 10^1^	4.3403 × 10^3^	9.9910 × 10^2^	5.1451 × 10^3^	6.8974 × 10^2^
SMA	9.6968 × 10^2^	2.8388 × 10^1^	5.7536 × 10^3^	9.0981 × 10^2^	5.6918 × 10^3^	6.1943 × 10^2^
BA	1.0441 × 10^3^	4.9251 × 10^1^	1.2690 × 10^4^	4.6140 × 10^3^	5.5111 × 10^3^	8.1083 × 10^2^
FA	1.0523 × 10^3^	1.1106 × 10^1^	5.3093 × 10^3^	5.7708 × 10^2^	8.1169 × 10^3^	3.0811 × 10^2^
	F10		F11		F12	
	Avg	Std	Avg	Std	Avg	Std
CCMGO	1.1777 × 10^3^	2.7442 × 10^1^	7.6021 × 10^5^	6.7582 × 10^5^	2.3250 × 10^4^	1.3882 × 10^4^
MGO	1.1888 × 10^3^	2.4376 × 10^1^	7.8080 × 10^5^	5.0623 × 10^5^	3.0908 × 10^4^	2.6628 × 10^4^
WOA	1.4723 × 10^3^	7.1039 × 10^1^	4.3190 × 10^7^	2.9598 × 10^7^	1.3858 × 10^5^	7.4986 × 10^4^
GWO	1.9076 × 10^3^	9.3785 × 10^2^	7.4988 × 10^7^	1.0179 × 10^8^	4.8309 × 10^6^	2.5806 × 10^7^
MFO	5.0242 × 10^3^	5.8710 × 10^3^	5.5507 × 10^8^	6.3529 × 10^8^	1.1620 × 10^8^	3.2070 × 10^8^
SCA	2.1162 × 10^3^	3.1725 × 10^2^	1.1021 × 10^9^	2.6941 × 10^8^	3.8766 × 10^8^	1.7146 × 10^8^
PSO	1.1985 × 10^3^	2.3743 × 10^1^	3.3605 × 10^4^	1.8907 × 10^4^	1.6430 × 10^4^	1.5195 × 10^4^
SMA	1.5378 × 10^3^	9.6734 × 10^1^	9.1920 × 10^7^	4.5310 × 10^7^	2.1682 × 10^6^	2.3372 × 10^6^
BA	1.2941 × 10^3^	5.9040 × 10^1^	2.5194 × 10^6^	1.9557 × 10^6^	3.1477 × 10^5^	1.2367 × 10^5^
FA	3.4537 × 10^3^	4.6384 × 10^2^	1.5031 × 10^9^	2.7479 × 10^8^	5.5605 × 10^8^	1.5926 × 10^8^
	F13		F14		F15	
	Avg	Std	Avg	Std	Avg	Std
CCMGO	6.6078 × 10^3^	4.2189 × 10^3^	1.1788 × 10^4^	6.3326 × 10^3^	2.1765 × 10^3^	1.3227 × 10^2^
MGO	1.5084 × 10^4^	9.8346 × 10^3^	2.2119 × 10^4^	1.8794 × 10^4^	2.1519 × 10^3^	1.6588 × 10^2^
WOA	8.7090 × 10^5^	9.4998 × 10^5^	6.6626 × 10^4^	3.6297 × 10^4^	3.4919 × 10^3^	4.3085 × 10^2^
GWO	2.2354 × 10^5^	4.4007 × 10^5^	2.0655 × 10^5^	5.1690 × 10^5^	2.3664 × 10^3^	2.7197 × 10^2^
MFO	4.3377 × 10^5^	1.2584 × 10^6^	3.0167 × 10^7^	1.6486 × 10^8^	3.1544 × 10^3^	3.3005 × 10^2^
SCA	1.4238 × 10^5^	7.9330 × 10^4^	1.2004 × 10^7^	1.0574 × 10^7^	3.6654 × 10^3^	1.8768 × 10^2^
PSO	7.4037 × 10^3^	4.8823 × 10^3^	6.3968 × 10^3^	6.1663 × 10^3^	2.8344 × 10^3^	3.6717 × 10^2^
SMA	1.6322 × 10^5^	9.4226 × 10^4^	1.7638 × 10^4^	6.0717 × 10^3^	2.8870 × 10^3^	3.1743 × 10^2^
BA	6.3676 × 10^3^	3.0735 × 10^3^	1.2503 × 10^5^	9.6707 × 10^4^	3.3392 × 10^3^	4.9046 × 10^2^
FA	2.3415 × 10^5^	1.0609 × 10^5^	6.1143 × 10^7^	2.9214 × 10^7^	3.4202 × 10^3^	1.7189 × 10^2^
	F16		F17		F18	
	Avg	Std	Avg	Std	Avg	Std
CCMGO	1.8418 × 10^3^	4.7839 × 10^1^	1.8777 × 10^5^	1.0793 × 10^5^	8.0208 × 10^3^	5.0355 × 10^3^
MGO	1.8872 × 10^3^	5.3940 × 10^1^	3.0783 × 10^5^	2.1066 × 10^5^	1.5241 × 10^4^	1.1343 × 10^4^
WOA	2.5695 × 10^3^	2.6375 × 10^2^	2.9573 × 10^6^	3.3986 × 10^6^	3.4772 × 10^6^	2.2369 × 10^6^
GWO	1.9990 × 10^3^	1.5469 × 10^2^	6.8374 × 10^5^	8.2071 × 10^5^	4.8725 × 10^5^	6.5325 × 10^5^
MFO	2.5272 × 10^3^	3.1294 × 10^2^	2.3283 × 10^6^	4.6075 × 10^6^	1.6749 × 10^7^	3.8286 × 10^7^
SCA	2.4031 × 10^3^	1.5192 × 10^2^	2.8800 × 10^6^	1.6831 × 10^6^	2.3285 × 10^7^	1.1446 × 10^7^
PSO	2.4411 × 10^3^	3.0918 × 10^2^	1.2966 × 10^5^	8.1880 × 10^4^	1.0706 × 10^4^	8.8187 × 10^3^
SMA	2.2685 × 10^3^	2.0445 × 10^2^	6.7114 × 10^5^	6.1655 × 10^5^	4.2415 × 10^5^	5.4044 × 10^5^
BA	2.8397 × 10^3^	2.6454 × 10^2^	1.7296 × 10^5^	1.4578 × 10^5^	6.3761 × 10^5^	2.8491 × 10^5^
FA	2.5193 × 10^3^	1.3317 × 10^2^	4.0789 × 10^6^	1.7465 × 10^6^	9.8337 × 10^7^	3.0392 × 10^7^
	F19		F20		F21	
	Avg	Std	Avg	Std	Avg	Std
CCMGO	2.2133 × 10^3^	7.2694 × 10^1^	2.3522 × 10^3^	1.0086 × 10^1^	2.8794 × 10^3^	1.1681 × 10^3^
MGO	2.2436 × 10^3^	8.0062 × 10^1^	2.3582 × 10^3^	3.2713 × 10^1^	2.9752 × 10^3^	1.3490 × 10^3^
WOA	2.7923 × 10^3^	1.7679 × 10^2^	2.5710 × 10^3^	6.1411 × 10^1^	6.2553 × 10^3^	1.9373 × 10^3^
GWO	2.3771 × 10^3^	1.5767 × 10^2^	2.3791 × 10^3^	1.9638 × 10^1^	4.6370 × 10^3^	1.6141 × 10^3^
MFO	2.6655 × 10^3^	2.1973 × 10^2^	2.4983 × 10^3^	4.3194 × 10^1^	6.3968 × 10^3^	1.5134 × 10^3^
SCA	2.5862 × 10^3^	1.1281 × 10^2^	2.5561 × 10^3^	2.0667 × 10^1^	8.9634 × 10^3^	1.6339 × 10^3^
PSO	2.6441 × 10^3^	2.1825 × 10^2^	2.4721 × 10^3^	4.4254 × 10^1^	4.9468 × 10^3^	2.2506 × 10^3^
SMA	2.4495 × 10^3^	1.4614 × 10^2^	2.4708 × 10^3^	2.4979 × 10^1^	4.0565 × 10^3^	2.1651 × 10^3^
BA	3.0151 × 10^3^	2.8197 × 10^2^	2.6002 × 10^3^	4.7096 × 10^1^	6.8688 × 10^3^	1.7185 × 10^3^
FA	2.6088 × 10^3^	8.7682 × 10^1^	2.5384 × 10^3^	1.4015 × 10^1^	3.8333 × 10^3^	1.2889 × 10^2^
	F22		F23		F24	
	Avg	Std	Avg	Std	Avg	Std
CCMGO	2.7098 × 10^3^	2.2201 × 10^1^	2.8826 × 10^3^	1.1959 × 10^1^	2.8869 × 10^3^	9.6118 × 10^−1^
MGO	2.7200 × 10^3^	1.2828 × 10^1^	2.8934 × 10^3^	1.3821 × 10^1^	2.8873 × 10^3^	6.4718 × 10^−1^
WOA	3.0563 × 10^3^	8.1959 × 10^1^	3.1756 × 10^3^	8.7993 × 10^1^	2.9499 × 10^3^	3.8644 × 10^1^
GWO	2.7476 × 10^3^	3.3271 × 10^1^	2.9316 × 10^3^	4.7673 × 10^1^	2.9811 × 10^3^	3.6181 × 10^1^
MFO	2.8323 × 10^3^	3.9181 × 10^1^	2.9938 × 10^3^	3.4765 × 10^1^	3.3149 × 10^3^	4.5989 × 10^2^
SCA	2.9828 × 10^3^	2.9394 × 10^1^	3.1635 × 10^3^	2.4587 × 10^1^	3.2066 × 10^3^	5.3344 × 10^1^
PSO	3.3244 × 10^3^	1.2112 × 10^2^	3.3639 × 10^3^	1.8302 × 10^2^	2.8813 × 10^3^	1.2226 × 10^1^
SMA	2.8518 × 10^3^	3.4349 × 10^1^	3.0024 × 10^3^	2.1021 × 10^1^	3.0105 × 10^3^	4.2694 × 10^1^
BA	3.3377 × 10^3^	1.4683 × 10^2^	3.3551 × 10^3^	1.3067 × 10^2^	2.9097 × 10^3^	2.2350 × 10^1^
FA	2.9113 × 10^3^	1.0986 × 10^1^	3.0672 × 10^3^	1.2063 × 10^1^	3.5529 × 10^3^	1.0610 × 10^2^
	F25		F26		F27	
	Avg	Std	Avg	Std	Avg	Std
CCMGO	4.0360 × 10^3^	4.4910 × 10^2^	3.2084 × 10^3^	5.6067 × 10^0^	3.2191 × 10^3^	1.1333 × 10^1^
MGO	4.1057 × 10^3^	4.4247 × 10^2^	3.2110 × 10^3^	5.7906 × 10^0^	3.2263 × 10^3^	1.2693 × 10^1^
WOA	7.1806 × 10^3^	9.9826 × 10^2^	3.3544 × 10^3^	9.4240 × 10^1^	3.2966 × 10^3^	3.3966 × 10^1^
GWO	4.5703 × 10^3^	3.1382 × 10^2^	3.2494 × 10^3^	2.6475 × 10^1^	3.4278 × 10^3^	7.1809 × 10^1^
MFO	6.1219 × 10^3^	4.4450 × 10^2^	3.2484 × 10^3^	2.2024 × 10^1^	4.2467 × 10^3^	7.9835 × 10^2^
SCA	6.9624 × 10^3^	3.2108 × 10^2^	3.4048 × 10^3^	4.8784 × 10^1^	3.8418 × 10^3^	1.3263 × 10^2^
PSO	6.8713 × 10^3^	2.1881 × 10^3^	3.3191 × 10^3^	3.3374 × 10^2^	3.1604 × 10^3^	5.7726 × 10^1^
SMA	5.1885 × 10^3^	6.1139 × 10^2^	3.2600 × 10^3^	3.0310 × 10^1^	3.4233 × 10^3^	4.0169 × 10^1^
BA	9.0997 × 10^3^	2.1628 × 10^3^	3.4647 × 10^3^	1.4833 × 10^2^	3.1460 × 10^3^	5.8797 × 10^1^
FA	6.5233 × 10^3^	1.4455 × 10^2^	3.3370 × 10^3^	1.4475 × 10^1^	3.8857 × 10^3^	8.5131 × 10^1^
	F28		F29			
	Avg	Std	Avg	Std		
CCMGO	3.6150 × 10^3^	8.3397 × 10^1^	4.3106 × 10^4^	2.7155 × 10^4^		
MGO	3.6077 × 10^3^	6.6509 × 10^1^	7.3720 × 10^4^	4.3047 × 10^4^		
WOA	4.6753 × 10^3^	3.6858 × 10^2^	1.1157 × 10^7^	7.7763 × 10^6^		
GWO	3.7680 × 10^3^	1.7323 × 10^2^	5.9954 × 10^6^	8.1820 × 10^6^		
MFO	4.1237 × 10^3^	2.7095 × 10^2^	1.3106 × 10^6^	3.7924 × 10^6^		
SCA	4.5999 × 10^3^	1.9125 × 10^2^	7.0834 × 10^7^	2.8824 × 10^7^		
PSO	4.0102 × 10^3^	3.2291 × 10^2^	5.2893 × 10^3^	2.9526 × 10^3^		
SMA	4.0647 × 10^3^	2.2025 × 10^2^	6.6360 × 10^6^	4.7522 × 10^6^		
BA	5.0038 × 10^3^	4.1198 × 10^2^	9.7148 × 10^5^	6.7934 × 10^5^		
FA	4.6803 × 10^3^	1.5299 × 10^2^	1.0007 × 10^8^	2.3892 × 10^7^		
	Overall Rank					
	RANK	+/=-	AVG			
CCMGO	1	~	1.6207			
MGO	2	18/10/1	2.6552			
WOA	8	29/0/0	7.4828			
GWO	4	29/0/0	4.4828			
MFO	7	29/0/0	7.0000			
SCA	9	29/0/0	7.9310			
PSO	3	16/4/9	3.7931			
SMA	5	29/0/0	5.3448			
BA	6	24/2/3	6.6552			
FA	10	29/0/0	8.0345			

**Table 5:** The *p*-values of the CCMGO versus the other algorithms on CEC2017.

	CCMGO	MGO	WOA	GWO	MFO
F1	/	1.20 × 10^−1^	1.73 × 10^−6^	1.73 × 10^−6^	1.73 × 10^−6^
F2	/	1.73 × 10^−6^	1.73 × 10^−6^	1.73 × 10^−6^	4.73 × 10^−6^
F3	/	3.09 × 10^−1^	1.73 × 10^−6^	1.92 × 10^−6^	1.92 × 10^−6^
F4	/	2.35 × 10^−6^	1.73 × 10^−6^	1.73 × 10^−6^	1.73 × 10^−6^
F5	/	8.31 × 10^−4^	1.73 × 10^−6^	1.73 × 10^−6^	1.73 × 10^−6^
F6	/	4.29 × 10^−6^	1.73 × 10^−6^	1.73 × 10^−6^	1.73 × 10^−6^
F7	/	1.49 × 10^−5^	1.73 × 10^−6^	2.35 × 10^−6^	1.73 × 10^−6^
F8	/	2.60 × 10^−6^	1.73 × 10^−6^	1.73 × 10^−6^	1.73 × 10^−6^
F9	/	5.22 × 10^−6^	1.73 × 10^−6^	2.56 × 10^−2^	1.92 × 10^−6^
F10	/	3.87 × 10^−2^	1.73 × 10^−6^	1.73 × 10^−6^	1.73 × 10^−6^
F11	/	7.81 × 10^−1^	1.73 × 10^−6^	1.73 × 10^−6^	1.73 × 10^−6^
F12	/	3.60 × 10^−1^	1.73 × 10^−6^	3.88 × 10^−6^	3.52 × 10^−6^
F13	/	4.90 × 10^−4^	1.73 × 10^−6^	1.73 × 10^−6^	1.73 × 10^−6^
F14	/	1.71 × 10^−3^	1.73 × 10^−6^	1.73 × 10^−6^	2.35 × 10^−6^
F15	/	7.34 × 10^−1^	1.92 × 10^−6^	2.11 × 10^−3^	1.73 × 10^−6^
F16	/	6.84 × 10^−3^	1.73 × 10^−6^	1.64 × 10^−5^	1.73 × 10^−6^
F17	/	2.56 × 10^−2^	3.18 × 10^−6^	4.20 × 10^−4^	7.71 × 10^−4^
F18	/	7.27 × 10^−3^	1.73 × 10^−6^	1.73 × 10^−6^	2.60 × 10^−6^
F19	/	1.47 × 10^−1^	1.73 × 10^−6^	1.48 × 10^−4^	1.92 × 10^−6^
F20	/	2.18 × 10^−2^	1.73 × 10^−6^	4.45 × 10^−5^	1.73 × 10^−6^
F21	/	4.95 × 10^−2^	4.73 × 10^−6^	3.72 × 10^−5^	6.34 × 10^−6^
F22	/	1.57 × 10^−2^	1.73 × 10^−6^	2.60 × 10^−5^	1.92 × 10^−6^
F23	/	5.67 × 10^−3^	1.73 × 10^−6^	5.22 × 10^−6^	1.73 × 10^−6^
F24	/	8.59 × 10^−2^	1.73 × 10^−6^	1.73 × 10^−6^	1.92 × 10^−6^
F25	/	6.29 × 10^−1^	1.92 × 10^−6^	1.24 × 10^−5^	1.73 × 10^−6^
F26	/	1.31 × 10^−1^	1.73 × 10^−6^	1.92 × 10^−6^	1.73 × 10^−6^
F27	/	3.00 × 10^−2^	1.73 × 10^−6^	1.73 × 10^−6^	1.73 × 10^−6^
F28	/	9.59 × 10^−1^	1.73 × 10^−6^	2.22 × 10^−4^	2.35 × 10^−6^
F29	/	1.38 × 10^−3^	1.73 × 10^−6^	1.73 × 10^−6^	1.80 × 10^−5^
	SCA	PSO	SMA	BA	FA
F1	1.73 × 10^−6^	4.73 × 10^−6^	1.73 × 10^−6^	5.22 × 10^−6^	1.73 × 10^−6^
F2	1.73 × 10^−6^	1.73 × 10^−6^	1.73 × 10^−6^	1.73 × 10^−6^	1.73 × 10^−6^
F3	1.73 × 10^−6^	3.18 × 10^−6^	1.73 × 10^−6^	1.48 × 10^−2^	1.73 × 10^−6^
F4	1.73 × 10^−6^	1.73 × 10^−6^	1.73 × 10^−6^	1.73 × 10^−6^	1.73 × 10^−6^
F5	1.73 × 10^−6^	1.73 × 10^−6^	1.73 × 10^−6^	1.73 × 10^−6^	1.73 × 10^−6^
F6	1.73 × 10^−6^	1.73 × 10^−6^	1.73 × 10^−6^	1.73 × 10^−6^	1.73 × 10^−6^
F7	1.73 × 10^−6^	1.73 × 10^−6^	1.73 × 10^−6^	1.73 × 10^−6^	1.73 × 10^−6^
F8	1.73 × 10^−6^	1.73 × 10^−6^	1.73 × 10^−6^	1.73 × 10^−6^	1.73 × 10^−6^
F9	1.73 × 10^−6^	4.29 × 10^−6^	1.92 × 10^−6^	1.73 × 10^−6^	1.73 × 10^−6^
F10	1.73 × 10^−6^	1.40 × 10^−2^	1.73 × 10^−6^	1.73 × 10^−6^	1.73 × 10^−6^
F11	1.73 × 10^−6^	1.73 × 10^−6^	1.73 × 10^−6^	7.69 × 10^−6^	1.73 × 10^−6^
F12	1.73 × 10^−6^	9.37 × 10^−2^	1.73 × 10^−6^	1.73 × 10^−6^	1.73 × 10^−6^
F13	1.73 × 10^−6^	6.00 × 10^−1^	1.73 × 10^−6^	6.58 × 10^−1^	1.73 × 10^−6^
F14	1.73 × 10^−6^	5.32 × 10^−3^	1.11 × 10^−3^	1.73 × 10^−6^	1.73 × 10^−6^
F15	1.73 × 10^−6^	2.60 × 10^−6^	1.92 × 10^−6^	1.73 × 10^−6^	1.73 × 10^−6^
F16	1.73 × 10^−6^	1.92 × 10^−6^	2.35 × 10^−6^	1.73 × 10^−6^	1.73 × 10^−6^
F17	1.73 × 10^−6^	1.96 × 10^−2^	2.22 × 10^−4^	4.17 × 10^−1^	1.73 × 10^−6^
F18	1.73 × 10^−6^	5.04 × 10^−1^	3.52 × 10^−6^	1.73 × 10^−6^	1.73 × 10^−6^
F19	1.73 × 10^−6^	1.73 × 10^−6^	2.35 × 10^−6^	1.73 × 10^−6^	1.73 × 10^−6^
F20	1.73 × 10^−6^	1.73 × 10^−6^	1.73 × 10^−6^	1.73 × 10^−6^	1.73 × 10^−6^
F21	1.73 × 10^−6^	2.58 × 10^−3^	5.71 × 10^−4^	3.88 × 10^−6^	1.48 × 10^−4^
F22	1.73 × 10^−6^	1.73 × 10^−6^	1.73 × 10^−6^	1.73 × 10^−6^	1.73 × 10^−6^
F23	1.73 × 10^−6^	2.60 × 10^−6^	1.73 × 10^−6^	1.73 × 10^−6^	1.73 × 10^−6^
F24	1.73 × 10^−6^	8.94 × 10^−4^	1.73 × 10^−6^	1.02 × 10^−5^	1.73 × 10^−6^
F25	1.73 × 10^−6^	1.36 × 10^−5^	4.29 × 10^−6^	2.35 × 10^−6^	1.73 × 10^−6^
F26	1.73 × 10^−6^	5.71 × 10^−2^	1.73 × 10^−6^	1.73 × 10^−6^	1.73 × 10^−6^
F27	1.73 × 10^−6^	6.32 × 10^−5^	1.73 × 10^−6^	2.84 × 10^−5^	1.73 × 10^−6^
F28	1.73 × 10^−6^	3.88 × 10^−6^	1.92 × 10^−6^	1.73 × 10^−6^	1.73 × 10^−6^
F29	1.73 × 10^−6^	1.92 × 10^−6^	1.73 × 10^−6^	1.73 × 10^−6^	1.73 × 10^−6^

**Table 6:** Properties of the three-channel model.

Properties	Value
Grid Size	25 × 25 × 1
Depth	4800 ft
Initial Pressure	4000 psi
Porosity	0.2
Compressibility	6.9 × 10^−5^ psi^−1^
Initial Water Saturation	0.2
Viscosity	2.2 cP

**Table 7:** The results of CCMGO and the other algorithms on the oil reservoir production optimization.

Algorithm	NPV (USD)			
	Mean	Std	Best	Worst
CCMGO	9.4969 × 10^7^	1.7636 × 10^6^	9.7758 × 10^7^	9.2169 × 10^7^
MGO	9.3972 × 10^7^	2.2134 × 10^6^	9.8885 × 10^7^	9.0026 × 10^7^
MFO	8.9384 × 10^7^	3.2705 × 10^6^	9.5890 × 10^7^	8.4333 × 10^7^
GWO	9.0767 × 10^7^	3.8653 × 10^6^	9.8620 × 10^7^	8.3161 × 10^7^
PSO	8.2722 × 10^7^	1.9501 × 10^6^	8.6192 × 10^7^	7.7980 × 10^7^
SMA	9.0067 × 10^7^	2.9355 × 10^6^	9.6942 × 10^7^	8.4874 × 10^7^
WOA	8.6832 × 10^7^	2.8288 × 10^6^	9.2802 × 10^7^	8.0419 × 10^7^

## Data Availability

The raw data supporting the conclusions of this article will be made available by the authors on request.
